# Congenital Cytomegalovirus infection: advances and challenges in diagnosis, prevention and treatment

**DOI:** 10.1186/s13052-017-0358-8

**Published:** 2017-04-17

**Authors:** Concetta Marsico, David W. Kimberlin

**Affiliations:** 1 Department of Medical and Surgical Sciences, Neonatology and Neonatal Intensive Care Unit, St.Orsola-Malpighi Hospital, University of Bologna, via Massarenti 11, 40138 Bologna, Italy; 2 Department of Pediatrics, Division of Pediatric Infectious Diseases, University of Alabama at Birmingham, Children’s Harbor Building 303, 1600 7th Avenue South, Birmingham, AL 35233 USA

**Keywords:** Congenital cytomegalovirus, Hearing loss, Mother-to-child transmission, Neonatal screening, Antiviral therapy

## Abstract

Cytomegalovirus (CMV) is the most frequent cause of congenital infection worldwide, with an estimated incidence in developing countries of 0.6–0.7% of all live births. The burden of disease related to congenital CMV in substantial, as it is the leading non-genetic cause of sensorineural hearing loss and an important cause of neurodevelopmental disabilities in children. Despite its clinical significance, congenital CMV infection often goes undetected because the majority of infected infants are asymptomatic at birth and screening programs have not been substantially implemented. Other than behavioral measures, effective interventions aimed at the prevention of maternal infection and of mother-to-child transmission are lacking. Due to a convergence of recent advances in both diagnostic and therapeutic strategies in infants with congenital CMV, though, the field likely will be changing rapidly over just the next few years. Specifically, a highly-sensitive screening test with high throughput potential has been developed, and treatment of infants symptomatically infected with congenital CMV has proven to be well-tolerated and effective in improving long-term hearing and neurodevelopmental outcomes.

This review highlights the clinical importance of congenital CMV infection, the developments in laboratory diagnostics, and the benefits of antiviral therapy. It also identifies the global efforts still required in the prevention of maternal infection and in the optimization of antiviral therapy to further reduce the burden of congenital CMV disease.

## Background

Human Cytomegalovirus (CMV) is a ubiquitous human-specific DNA virus, belonging to the *Herpesviridae* family. The vast majority of CMV infections are asymptomatic or self-limited in healthy children and adults. In immunocompromised hosts and infected fetuses, though, CMV produces a high burden of disease. Congenital CMV infection is the most common congenital infection worldwide, and in the developed world it is the leading non-genetic cause of sensorineural hearing loss (SNHL) in children and an important cause of neurodevelopmental delay [1–3].

This review highlights the clinical significance of congenital CMV infection, the advances in the diagnostic techniques that can have direct implications for the development and implementation of screening programs, and the available therapeutic strategies to prevent mother-to-child transmission (MTCT) and to improve long-term outcomes of symptomatic infants.

## Clinical significance of congenital CMV infection

CMV infection is endemic and does not show seasonal variations [4]. The CMV seroprevalence varies widely among populations and is characterized by an age-dependent rise. In developed countries, CMV seroprevalence in women of child-bearing age ranges from less than 50 to 85%, while in developing countries where infection generally is acquired very early in life through breastfeeding and crowded living conditions the seroprevalence approximates 100%. Additional factors associated with higher seroprevalence are lower socioeconomic levels, nonwhite races, caring for young children, and sexual activity [5–7].

Intrauterine CMV transmission may occur in mothers without preexisting immunity who first acquire CMV infection in pregnancy (primary infection), or in women with preexisting antibodies to CMV either by reactivation of a previous maternal infection or by acquisition of a different viral strain (non-primary infection) [8]. Primary CMV infections are associated with the greatest risk of in-utero transmission at 30–35%, while for non-primary infections the transmission rate is significantly lower at 1.1–1.7% [1]. Because of the high CMV seroprevalence in adults, congenital CMV infection results from non-primary maternal infections in nearly two-thirds of infected infants [9, 10]. The rate of vertical transmission increases with older gestational age at infection, while there is a higher risk of fetal damage when infection occurs in the early stages of pregnancy [11–14].

Congenital CMV infection is the most common congenital infection worldwide, with an estimated incidence in developed countries that ranges from 0.6 to 0.7% of all live births, resulting in approximately 60,000 neonates born every year with congenital CMV infection in the United States and the European Union combined [1, 2, 15]. Since the incidence of congenital CMV infection parallels maternal seroprevalence, the estimated incidence in developing countries is even higher, between 1 and 5% of all live births [5, 6].

The clinical spectrum of congenital CMV infection varies widely, from the complete absence of signs of infection (asymptomatic infection) to potentially life-threatening disseminated disease. At birth, 85–90% of infected infants are asymptomatic, and 10–15% present with clinical apparent infection (symptomatic disease) [1, 2]. The presentation in this latter group is a continuum of disease expression whose more common findings are petechiae, jaundice, hepatomegaly, splenomegaly, microcephaly, and other neurologic signs. Laboratory and imaging findings include thrombocytopenia, transaminitis, direct hyperbilirubinemia, chorioretinitis, neuroimaging abnormalities indicative of central nervous system (CNS) involvement, and SNHL [16, 17]. However, the diagnostic criteria of symptomatic infection vary widely in the literature. For instance, some case series consider subjects with abnormalities detected by using specific testing, including SNHL, as asymptomatic, while others do not [18, 19]. And some studies have categorized infants with isolated low birth weight as symptomatic, whereas others have not [3, 17]. These differences may account to some of the variability in the prevalence of symptomatic infection and of disease severity across studies.

Among infants who develop CMV-related SNHL, hearing loss may be present at birth or may be delayed in onset. Late-onset SNHL occurs throughout the first several years of life, with a median age at onset of 33 months for symptomatic and 44 months for asymptomatic infants. Approximately 50% of children with SNHL have further deterioration or progression of their loss during childhood, and the degree of hearing loss may fluctuate in up to half of infants [20]. Therefore, it is important that all infants with congenital CMV infection, irrespective from their clinical presentation at birth, receive serial audiological monitoring throughout the first years of life to allow for early detection of possible SNHL. Even if no specific pharmacological therapy can be offered to children who develop CMV-related SNHL, early identification and non-pharmacological interventions can reduce the functional impairment resulting from hearing loss, significantly improving the receptive and expressive language and also the social-emotional development of the affected child [21, 22].

Overall nearly 50% of symptomatic and 10% of asymptomatic infants develop some degree of hearing loss, making congenital CMV infection the leading non-genetic cause of SNHL in children [2, 3]. It is estimated that almost 25% of hearing loss in children of 4 years of age is attributable to congenital CMV [23]. Vestibular impairment also has been reported frequently, and possibly can show progressive deterioration over time [24]. Furthermore, congenital CMV is the leading viral cause of neurodevelopmental delay, with a large proportion of symptomatic infants suffering of some degree of psychomotor and cognitive disabilities, and with visual impairment in up to half of symptomatic infants [2, 17, 25].

As many affected children require significant ongoing care and special therapeutic and educational services, the economic burden associated to congenital CMV infection is substantial: in the United States the estimated annual cost of congenital CMV infection is up to $2 billion in 1992 dollars (which correlates with in excess of $3 billion today), which contributed to the identification of the need for a CMV vaccine as a high priority for the 21^st^ century [26].

## Diagnosis of congenital CMV infection

Despite its health, social, and economic burden, congenital CMV infection often goes undetected at birth because the majority of affected infants are asymptomatic or present with symptoms that are sufficiently nonspecific that they do not prompt clinicians to suspect CMV infection. Screening programs, both in pregnant women and in newborns, have not been developed or implemented. In the past years one of the obstacles to the implementation of a neonatal screening program has been the lack of a screening test well-suited for high-throughput analyses.

Traditionally, virus isolation from urine or saliva in tissue cultures has been the standard method for diagnosing congenital CMV infection. This technique is labor- and resource-intensive, requires tissue cultures, and thus is not suitable for widespread screening purposes. The optimization of the real-time polymerase chain reaction (PCR) technology has led to important advances in the diagnostic possibilities, since it is amenable to automation, is low-cost, and is unlikely to be affected by sample storage and transport conditions [27, 28].

In view of the routes of metabolic and genetic screening programs existing in many countries, dried blood spots (DBS) have been hypothesized as a practical screening specimen for congenital CMV infection. The only large, population-based, prospective comparison of CMV DNA detection by real-time PCR in DBS to a culture-based assay in congenital CMV infection was conducted by the National Institute on Deafness and Other Communication Disorders (NIDCD) CMV and Hearing Multicenter Screening (CHIMES) Study. In this trial, DBS PCR assays produced disappointing results [27]. Two different DNA extraction and PCR protocols were used to determine sensitivity and specificity of DBS real-time PCR assays. Of the 20,448 screened infants, 92 (0.45%) had confirmed congenital CMV infection, but only 28 of these 92 infected infants (30%) were diagnosed with congenital CMV infection by using DBS real-time PCR assays. Overall, the sensitivity of DBS PCR ranged from 28.3% (95% confidence interval [CI], 17.4% – 41.4%) using a single-primer assay to 34.4% (95% CI, 18.6% – 53.2%) using a two-primer assay. The specificity of both PCR protocols was 99.9% (95% CI, 99.9% – 100%). Other studies reported variably higher sensitivity of CMV DNA detection by PCR in DBS, up to 100%, but all were retrospective studies or prospective studies of selected populations with known CMV infection [29–33]. The reasons of this great variability in sensitivity might be related to technical issues (DNA extraction method, PCR protocol, amount of the spotted paper), but also could be due to the fact that not all infants with congenital CMV infection, either symptomatic or asymptomatic, have detectable viremia at birth [34].

At this time and with the available methods, CMV testing with DBS real-time PCR therefore is unsuitable for the purpose of CMV screening, and its main utility remains the retrospective diagnosis of congenital CMV infection in children who present with delayed-onset sequelae. In these situations, though, a positive result confirms congenital CMV infection but a negative result does not rule out congenital CMV infection.

Unlike the DBS specimens, real-time PCR assay on saliva swabs from the CHIMES study produced excellent results, both for air dried swabs and for swabs sent to the laboratory in viral transport medium [28]. The reported sensitivity and specificity of the liquid-saliva (those sent in viral transport media) PCR assay were 100% (95% CI, 95.8% to 100%) and 99.9% (95% CI, 99.9% to 100%), respectively; for the dried-saliva PCR assay sensitivity and specificity were 97.4% (95% CI, 90.8% to 99.7%) and 99.9% (95% CI, 99.9% to 100%), respectively. PCR assays were performed without a DNA extraction step, making this strategy even more practical for screening purposes. The rate of false positive results in both swabs was less than 0.03%; thus if saliva PCR assay is used to screen newborns, a positive screening result should be confirmed within the first 3 weeks of age to avoid false positive screening results [28, 35]. The excellent analytical sensitivity and the ease of saliva collection in neonates make this specimen advantageous for neonatal CMV screening. The other specimen in which CMV is constantly excreted in large amounts in congenital infection is urine, but its collection using a bag may be complicated in neonates by a number of factors (e.g., inadequate diuresis, loss of samples, contamination). The application of real-time PCR on urine collected by using other strategies, such as cotton balls or filter cards in diapers, has not been evaluated in large, population-based screening programs and has not been compared with a gold-standard diagnostic method [36, 37].

## Prevention of congenital CMV infection

Strategies to reduce the burden of congenital CMV disease may be implemented at different stages, and include prevention of maternal infection, prevention of MTCT, early detection and intervention by neonatal screening, and neonatal antiviral therapy.

Unlike many other infectious agents potentially harmful for the fetus and the neonate, prenatal screening by the use of maternal serology for CMV is not routinely recommended for several reasons, the most important being the unavailability of proven specific interventions for pregnant women who experience a primary CMV infection and also the fact that most congenitally infected babies are born to women experiencing a non-primary maternal infection. In 2005 a non-randomized study suggested that the administration of CMV-specific hyperimmune globulin (HIG) to pregnant women with primary CMV infection could lead to a significant decrease on the rate of MTCT (decreasing from 40 to 16%) and on the risk of congenital disease (decreasing from 50 to 3%) [38]. Subsequently, other non-randomized studies also showed improved outcomes in CMV-infected infants born to mothers who had received HIG in pregnancy, raising optimism around this strategy [39–41]. However, in 2014 results of the first phase II randomized, placebo-controlled trial on the use of virus-specific HIG for the prevention of congenital CMV infection were published and revealed that the difference in the rate of congenital infection between the group of pregnant women who had received HIG and the placebo group was not statistically significant (30% vs. 44%, *P* = 0.13) [42]. Because the effect was smaller than expected (14% percentage points), though, the power to detect such a difference with the available sample size (123 women) was also small. The study also showed that the clinical outcomes of congenital infection at birth were similar in the two groups, and that the number of obstetrical adverse events was higher in the HIG group as compared with the placebo group (13% vs. 2%). Two other phase III randomized, placebo-controlled trials of HIG in pregnancy for the prevention of CMV MTCT are currently ongoing in the United States (ClinicalTrials.gov, NCT01376778) and in Europe (ClinicalTrialsRegister.eu, EudraCT No. 2007-004692-19); both seek to enroll large numbers of pregnant women, which is proving challenging in the United States trial. Until those results are available, though, there is no controlled evidence supporting the use of HIG in pregnant women for the prevention or treatment of congenital CMV infection.

Antiviral therapy in women with CMV infection in pregnancy also has been studied in small series. Recently results of a phase II, multicenter, open-label study with one arm that evaluated the efficacy of high dose oral valacyclovir (8 g daily) in pregnant women carrying a moderately CMV infected fetus have been published [43]. A moderately CMV infected fetus was defined by the presence of one or more measurable extracerebral ultrasound features compatible with CMV infection and/or one isolated cerebral abnormality and/or laboratory findings of CMV infection in fetal blood. Women carrying fetuses without signs of infection and fetuses with severe ultrasound brain abnormalities were excluded. In this trial, 41 women received valacyclovir for a median of 89 days. The drug was well tolerated in this population. Using a Simon’s optimal two-stage design, valacyclovir was assumed to have a positive effect if at least 31/43 neonates were asymptomatic at birth. Study results showed that the number of asymptomatic infants at birth was 34/43, implying benefit of this therapeutic approach. Moreover, compared with a historical cohort, the use of valacyclovir significantly increased the proportion of asymptomatic neonates from 43% without treatment to 82% with treatment. Although these data are encouraging, given the limitation in both the sample size and the study design, this therapeutic strategy should be further investigated before antiviral therapy may be considered in pregnant women.

Another important limitation to prenatal screening for CMV is that, as mentioned above, a high percentage of neonates with congenital CMV infection is born to mothers with non-primary infections during pregnancy. Diagnosing a non-primary CMV infection in pregnancy is a challenge, since virological or immunological markers for non-primary CMV infections have not been yet identified [5].

This complex nature of CMV protective immunity, with the possibility of both reactivation of a previous infection and the risk of reinfection with genetically distinct viral strains, also produces a major challenge for the development of an effective CMV vaccine. An ideal vaccine aimed at reducing the impact of congenital CMV infection should have the ability to both protect seronegative women from primary infection, but also to augment the immune response in seropositive women to prevent reactivation or re-infection. Many CMV vaccine candidates are in different stages of investigation, and include adjuvanted recombinant protein vaccines based on the immunogenic capacity of the envelope glycoprotein B (gB) or on multicomponent subunit to produce a broader immunogenicity; recombinant live-attenuated replication-impaired or replication-defective vaccines; vaccines expressing immunogenic CMV gene products using DNA plasmid or peptide-based technologies; vectored vaccine approaches based on the expression of CMV antigens using live virus and virus-like particle (VLP) systems [44]. A previous phase II study of a gB/MF59 adjuvant subunit vaccine in postpartum women demonstrated an efficacy of approximately 50% against primary infection, with the protection observed predominantly in the first 12 months after vaccination [45]. In a subsequent assessment of samples from study, women who were enrolled and found to be seropositive also received the gB/MF59 vaccine or a placebo to determine if the vaccine produced an augmented antibody response; this investigation found that antibody titers in vaccinated women were boosted and remained higher 6 months after the final vaccine as compared with the placebo group [46]. Whether such boosting can prevent non-primary infection however is not known. Despite these observations and the many recent promising results of vaccine candidates in animal models and transplant recipients [44, 47], the prospect of a vaccine for the prevention of maternal and congenital infection does not appear feasible in the near future.

To date, the mainstay of interventions for the prevention of maternal infection, and in turn of congenital infection, remains the education of pregnant women regarding sources of exposure and behavioral interventions to limit exposure to CMV [35, 48]. A key source of CMV exposure is represented by young children who may shed CMV in saliva and urine. Therefore, specific behavioral guidance aimed at decreasing the transmission of CMV includes hand hygiene when caring for children, particularly after changing diapers or wiping a child’ nose, avoiding kissing children on their mouth and avoiding sharing food, drinks and other utensils that can be exposed to children’s bodily fluids. Studies have shown that these counselling-based interventions for CMV seronegative women may be effective in reducing CMV transmission [49, 50], and it is conceivable that limiting exposure to CMV could benefit also CMV seropositive women.

## Treatment of congenital CMV infection

The advances in neonatal antiviral therapy for symptomatic infants have been substantial in the last years. Studies assessing treatment of congenital CMV infection began 30 years ago, with ganciclovir (GCV, which is an acyclic deoxyguanosine analog) as the first drug to be evaluated.

In 2003 the Collaborative Antiviral Study Group (CASG) of the National Institute of Allergy and Infectious Diseases (NIAID) published the results of the first phase III, randomized, controlled trial of 6-week GCV therapy versus no treatment, to determine the effects of GCV on hearing function in symptomatic infants with CNS involvement [51]. One-hundred neonates, all ≤1 month of age, were enrolled and randomized to receive GCV at 6 mg/kg/dose intravenously twice daily or no treatment. The primary endpoint was hearing improvement, or for those with normal hearing at baseline preservation of normal hearing, between baseline and the 6-month follow-up. Even though the lost to follow-up rate in the study was high, results demonstrated that a 6-weeks GCV therapy improves hearing outcomes at 6 months. Indeed, 84% of GCV recipients had improved or protected hearing at 6 months, compared with 59% of the non-treated group (*P* = 0.06); none of the GCV recipients had hearing deterioration at 6 months, compared with 41% of the non-treated group (*P* < 0.01). Moreover 21% of GCV recipients had worsening in hearing in their best ear between baseline and 1 year or greater, as compared with 68% in the non-treated group (*P* = 0.002). The main toxicity related to this therapeutic strategy was the development of a clinically significant neutropenia in 63% of treated patients, as compared with 21% in the non-treated group (*P* < 0.01), as well as the need for a long-term intravenous access.

A subsequent pharmacokinetic/pharmacodynamic study by the CASG determined that 16 mg/kg/dose of valganciclovir (VGCV), the oral prodrug of GCV, given orally twice daily reliably provided comparable systemic exposure to GCV as achieved in the phase III trial of intravenous GCV, thereby avoiding the need of the intravenous access [34].

Based upon the fact that prolonged viral shedding occurs in congenitally infected infants, that they can present with delayed sequelae, and that CMV DNA has been detected in the perilymph of children undergoing cochlear implantation for CMV-induced SNHL up to 4 years of age [52], the CASG hypothesized that a longer duration of antiviral therapy could result in more prolonged suppression of viral replication and further improvement in outcomes. In the 2008–2013 period, the CASG conducted a phase III, randomized, placebo-controlled trial comparing 6 weeks of oral VGCV therapy (6-week group) with 6 months of oral VGCV therapy (6-month group) [19]. The study population consisted of symptomatic infants, all 30 days of age or less, with or without CNS involvement. This extension of the inclusion criteria, as compared with the first trial where only infants with CNS involvement were included, was based on the results of a follow-up study over a 30-year period suggesting that disseminated CMV disease at birth with or without CNS involvement was predictive of SNHL [53]. VGCV was administered at 16 mg/kg/dose twice daily. The primary end point of the study was the change in best-ear hearing from baseline to 6 months, and the secondary end points included the change in hearing from baseline to 12 and 24 months, and the comparison of neurodevelopmental outcome at 12 and 24 months by using the *Bayley Scales of Infant and Toddler Development*, III edition (Bayley-III), between the study groups. The study enrolled 109 infants, 96 of whom were assigned to receive blinded study medication after receiving 6 weeks of VGCV. Results showed that a longer duration of antiviral therapy does not further improve hearing function at 6 months, but improves hearing outcomes at 12 and 24 months as compared with a 6 weeks therapy (73% vs. 57%, *P* = 0.01; 77% vs. 64%, *P* = 0.04). At 24 months the 6-month group also had better neurodevelopmental scores on the language-composite component of the Bayley-III (*P* = 0.004), and there was suggestion of additional developmental benefits of therapy in the remaining components of the Bayley-III scores as well. Notably, oral VGCV was associated with a lower risk of neutropenia as compared with intravenous GCV: 19% of subjects developed a clinically significant neutropenia during the first 6 weeks of therapy, compared with 63% in the previous study of 6 weeks of intravenous GCV. After randomization in the more recent trial, the occurrence of neutropenia was similar between the 6-week and the 6-month group (21% vs. 27%, *P* = 0.64). Based on this study, VGCV for 6 months is now considered an effective and well-tolerated therapeutic option for symptomatic infants to improve hearing and neurodevelopmental long-term outcomes [35]. The decision to start antiviral therapy in infants with symptomatic congenital CMV infection should involve adequate counsel regarding the potential benefits and risks of antiviral therapy. Beside neutropenia, there is evidence of carcinogenicity and gonadotoxicity of GCV in some animal models, although no such toxicities have been demonstrated in humans at this time.

Despite these promising developments in antiviral therapy, some unanswered questions remain about the treatment of congenital CMV infection. Currently there is no evidence of benefit of antiviral therapy in asymptomatic infants, since they were not included in any of the above mentioned studies, and therefore asymptomatic infants should not receive antiviral therapy. A phase II trial of oral valganciclovir in this population will be started soon by the CASG. Since asymptomatic infants represent the vast majority of infants with congenital CMV infection and are at (an albeit lower as compared with symptomatic infants) risk of developing late-onset sequelae, many efforts have been undertaken to identify a neonatal predictor of SNHL in this population. If successful, the identification of a reliable biomarker to categorize asymptomatic infants into risk groups would importantly aid their management, both from a research and a clinical care perspective. Great attention has been focused on a potential predictive role of the viral load in peripheral blood, with some published studies (but not others) supporting this hypothesis [54–57]. Some of these studies reported a viral threshold above which the risk of SNHL seems to be increased [55, 56], while others have not. At the current time, viral load data in congenital CMV infection are not straightforward, and often are based on small numbers of subjects or lacking an adequate long-term follow-up. Further studies are urgently needed to clarify the utility of blood viral load as a predictor of SNHL. This point is of especially high importance in light of the renewed interest in screening programs for congenital CMV infection worldwide. It is likely that more asymptomatic infants will be identified by screening programs, thus leading to an urgent need of the understanding of the best management of these infants. Another important challenge in the application of the treatment data is whether antiviral treatment should be offered to infants with isolated, mild, aspecific findings, as well as to infants with isolated SNHL; infants with those characteristics were not enrolled in large numbers in the CASG’s clinical trials, and so there is no evidence on the efficacy of therapy in these infants. The last critical point is the management of children with isolated late-onset SNHL, and specifically whether antiviral therapy may be offered beyond the neonatal period to children who present or develop SNHL. Some of these points will hopefully be clarified in the next few years, since different trials including infants and children with CMV-related SNHL in whom treatment is started beyond the first month of life are ongoing. A phase III, non-randomized trial of 6-weeks VGCV is recruiting congenitally infected infants with isolated SNHL up to 12 weeks of age in the Netherlands, to evaluate the effect of VGCV in preventing the hearing deterioration at 20 months of age (ClinicalTrials.gov, NCT02005822). And a phase II, randomized, placebo-controlled trial of 6-weeks VGCV versus 6-weeks placebo by the CASG is recruiting children with SNHL from 1 to 48 months of age, to evaluate the change in the total ear hearing assessment (improved or no change versus others) from baseline to 6-months (ClinicalTrials.gov, NCT01649869).

## Conclusions

Congenital CMV infection is responsible of a high burden of disease worldwide. Preventive strategies other than behavioral measures during pregnancy are still lacking, but there is great research interest focused on the field. Significant advances in the diagnostic modalities and in neonatal antiviral therapies have occurred over the last years. A highly sensitive, wide-scale applicable screening test using an easily collected sample in neonates has been developed, making it feasible to consider developing and implementing a widespread screening program. An effective and well-tolerated antiviral therapy is now available for symptomatic infants to improve hearing and neurodevelopmental outcomes, even if it does not completely abolish the risk of long-term sequelae. No pharmacological options are available for asymptomatic infants and for infants with CMV-related morbidity beyond the neonatal period, but a large phase II study conducted by the CASG in asymptomatic infants will be starting soon in the United States. Ongoing efforts are aimed at providing evidence to support the decision-making process in these babies, with the final goal of improving the quality of life of the thousands of infants that each year are born with congenital CMV infection.

## Abbreviations

CASG: Collaborative Antiviral Study Group
CHIMES: CMV and Hearing Multicenter Screening
CMV: Cytomegalovirus
CNS: Central nervous system
DBS: Dried blood spots
GCV: Ganciclovir
HIG: Hyperimmune globulin
MTCT: Mother-to-child transmission
PCR: Polymerase chain reaction
SNHL: Sensorineural hearing loss
VGCV: Valganciclovir
